# Human iPSC-derived osteoblasts and osteoclasts together promote bone regeneration in 3D biomaterials

**DOI:** 10.1038/srep26761

**Published:** 2016-05-26

**Authors:** Ok Hee Jeon, Leelamma M. Panicker, Qiaozhi Lu, Jeremy J. Chae, Ricardo A. Feldman, Jennifer H. Elisseeff

**Affiliations:** 1 Translational Tissue Engineering Center, Wilmer Eye Institute and the Department of Biomedical Engineering, Johns Hopkins University, Baltimore, MD, USA; 2 Department of Microbiology and Immunology, University of Maryland School of Medicine, Baltimore, MD, USA; 3 Translational Tissue Engineering Center, Wilmer Eye Institute and the Department of Materials Science and Engineering, Johns Hopkins University, Baltimore, MD, USA

## Abstract

Bone substitutes can be designed to replicate physiological structure and function by creating a microenvironment that supports crosstalk between bone and immune cells found in the native tissue, specifically osteoblasts and osteoclasts. Human induced pluripotent stem cells (hiPSC) represent a powerful tool for bone regeneration because they are a source of patient-specific cells that can differentiate into all specialized cell types residing in bone. We show that osteoblasts and osteoclasts can be differentiated from hiPSC-mesenchymal stem cells and macrophages when co-cultured on hydroxyapatite-coated poly(lactic-*co*-glycolic acid)/poly(L-lactic acid) (HA–PLGA/PLLA) scaffolds. Both cell types seeded on the PLGA/PLLA especially with 5% w/v HA recapitulated the tissue remodeling process of human bone via coupling signals coordinating osteoblast and osteoclast activity and finely tuned expression of inflammatory molecules, resulting in accelerated *in vitro* bone formation. Following subcutaneous implantation in rodents, co-cultured hiPSC-MSC/-macrophage on such scaffolds showed mature bone-like tissue formation. These findings suggest the importance of coupling matrix remodeling through osteoblastic matrix deposition and osteoclastic tissue resorption and immunomodulation for tissue development.

Bone grafting is required to facilitate repair and regeneration of bone defects resulting from severe fracture in elderly osteoporosis patients, trauma, tumor ablation, or congenital abnormalities. Autologous grafts are regarded as the current gold standard, but due to their significant limitations bone tissue engineering has been explored as a promising alternative to autologous grafts^1^,^2^. Most of the current strategies in bone tissue engineering involve the cultivation of osteoblasts (OB) derived from human bone marrow mesenchymal stem cells (MSCs) on 3D alloplastic materials (primarily calcium phosphate, CaP) prior to re-implantation^3^. However, this approach fails to emulate physiological bone regeneration involving a well-orchestrated series of cytokines and regulatory molecules secreted from immune cells, including monocytes, macrophages, and osteoclasts (OC). An orchestrated performance of these molecules is required to mimic functional and structural intricacies of native bone, to integrate with the host environment, and to establish mechanical stability of the bone substitutes^4^,^5^. Thus, recreating the complex interplay between bone and immune cells in engineered substitutes may be necessary to create a functional tissue, rather than just to induce bone formation. We hypothesized that OBs and OCs—and their respective bone-building and -resorbing activities—must be balanced on bone-like synthetic scaffolds to create such bone substitutes.

Human induced pluripotent stem cells (hiPSC) constitute an exciting prospective cell source for engineering bone, as they can generate patient- or disease-specific mesenchymal and monocyte/macrophage precursors that in turn differentiate into OBs and OCs, respectively^6^. hiPSCs have been differentiated into OBs on 2D plastic and 3D CaP scaffolds^7^,^8^,^9^,^10^,^11^ and produced dense bone matrix on 3D decellurized bone scaffolds in a perfusion bioreactor^12^. OCs have been generated from hiPSCs on 2D plastic and on mineralized substrates by embryonic body (EB) formation^13^ and by co-culturing with stromal cells^14^. Nevertheless, to our knowledge, these derived OB and OC precursors have not been co-cultured to capture the functional coupling of bone resorption and formation that is typical of living bone.

Taking cues from natural bone tissue, the goal of this study was to engineer functional bone like-tissue constructs by co-culturing hiPSC-MSC and hiPSC-macrophages. Our previous work defined optimal scaffold compositions and tissue quality of bone engineered from human embryonic stem cells (hESC)^15^,^16^. In the present work, we first differentiated hiPSC-derived MSCs and macrophages into osteogenic and osteoclastogenic lineages, respectively, and established a 3D model of human bone by co-culturing these cells within HA-based scaffolds. We then explored bone formation and resorption *in vitro* and in a subcutaneous implantation model. We also studied molecular changes to identify the coupling signals and inflammatory molecules responsible for coordinating OB and OC activities.

Here we demonstrate that the incorporation of immune cells responsible for remodeling into a HA-based 3D co-culture model significantly improves bone formation *in vitro* and *in vivo* compared with mono-culture of osteogenic cells. Our data indicate that this improved bone formation likely results from the coordinated interaction of the two cell types, OB and OC, which are required for balanced bone remodeling through osteoprotegerin (OPG)/receptor activator of nuclear factor κB ligand (RANKL) and finely tuned expression of the inflammatory molecules interleukin 6 (IL-6), IL-1β, and tumor necrosis factor α (TNFα). This study offers new therapeutic approaches to treat bone defects by engineering personalized and functional bone substitutes that retain intrinsic osteogenic and remodeling capacity along with immunomodulatory signaling to promote regeneration.

## Results

### Differentiation of osteoblasts and osteoclasts from hiPSCs

hiPSCs cultured in suspension as EBs for 1 week differentiated into mesenchymal precursor cells after 10 days on gelatin-coated tissue culture plates. Cells that isolated from outgrowth of EBs and then expanded in monolayer culture with MSC growth medium exhibited typical fibroblastic cell morphology (Fig. S1A), expression of MSC surface markers CD44, CD73, and CD105 (Fig. S1B), and the potential to differentiate into three different cell linages: osteoblast (Fig. S1C), chondrocyte (Fig. S1D), and adipocyte (Fig. S1E). Under osteogenic conditions, hiPSC-MSCs displayed bone-specific alkaline phosphatase (ALP), calcium deposition, and up-regulation of runt-related transcription factor 2 (*RUNX2*), type I and X collagen (*COLI* and *COLX*), and osteocalcin (*OCN*) genes during 14 days of monolayer culture (Fig. S1C). hiPSC-MSCs grown for 28 days in 3D micromass culture or monolayer culture under chondrogenic or adipogenic conditions, respectively, produced proteoglycans and accumulated lipid with up-regulation of *SOX-9* and *Aggrecan* (Fig. S1D) as well as gene expression of fatty acid binding protein (*FABP*), lipoprotein lipase (*LPL*), and CCAAT-enhancer-binding proteins (*C/EBPα*) (Fig. S1E).

To induce OC from hiPSCs, we used a stepwise mono-culture approach based on our previous derivation strategy^17^ involving EB formation (Fig. S2A, i), direct differentiation to monocytes (Fig. S2A, ii and iii) and macrophages (Fig. S2A, iv), and differentiation into OCs (Fig. S2C). With this approach, hiPSCs cultured in suspension as EB for 10 days produced round, monocyte-like cells that arose from flattened EBs after 17 days with monocyte differentiation medium. Monocyte surface marker CD14 was expressed in more than 80% of collected cells floating in the culture medium (Fig. S2B). Monocytes were then differentiated into macrophages after plating onto adherent tissue culture plates for 5 days with macrophage differentiation medium supplemented with macrophage colony-stimulating factor (M-CSF) and IL-3. These cells had similar morphologies to spread-out macrophages (Fig. S2A, iv) and expressed macrophage markers CD14, CD68, and CD115 (Fig. S2B).

Next, hiPSC-derived macrophages were cultured in the presence of RANKL and M-CSF, which are essential cytokines for osteoclast differentiation and survival. Fourteen days after RANKL stimulation, multinucleated (>3 nuclei) and tartrate-resistant acid phosphatase (TRAP)–positive OCs were observed, as well as resorptive pits left by OCs (Fig. S2C). In hiPSC-derived OCs, cathepsin K colocalized with F-actin—a cysteine protease secreted by OCs during bone resorption and a characteristic feature of those cells to appose the bony surface for matrix digestion—, indicating functional OCs.

### Engineered 3D model of bone using both hiPSC-derived MSCs and macrophages

We developed a two-step culture approach to provide hiPSC-MSCs with a scaffold on which to differentiate into OBs and deposit bone like-matrix; then, OCs could be activated and survived. The goal was to co-culture hiPSC-MSCs and -macrophages committed to OB and OC lineages, respectively, on a HA-based 3D porous polyester composite scaffold. We reasoned that such a co-culture would mimic the *in vivo* bone environment and allow for natural crosstalk between bone cells.

**Figure 1:**
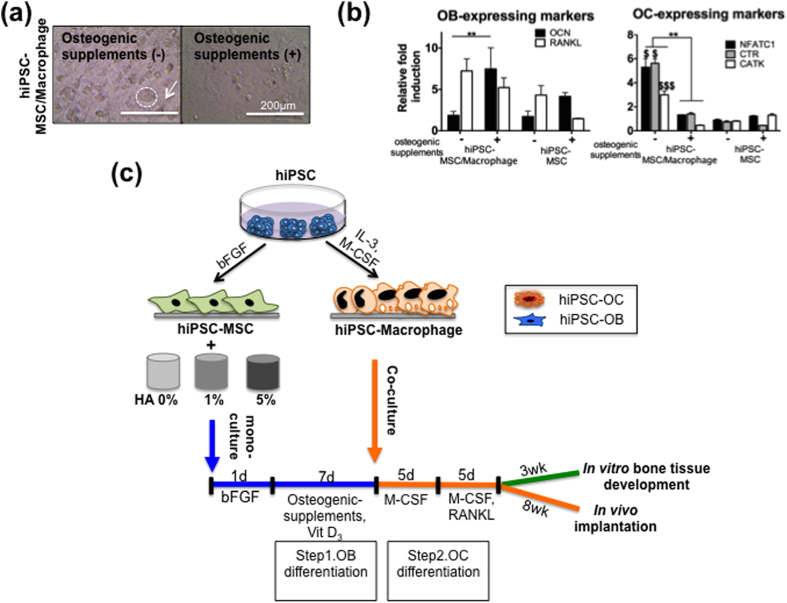
Engineered 3D model of bone using hiPSC-derived MSC and macrophage combination. (**a**) hiPSC-MSCs and -macrophages were co-cultured for 14 days with (+) or without (−) osteogenic supplements. Light microscopy images show multinucleated OCs (circles) embedded in an osteoblastic cell layer (arrow). Scale bars, 200 *μ*m. (**b**) Gene expression for OC-expressing markers, *NFATC1, CTR*, and *CATK* (Right) and OB-expressing markers *OCN* and *RANKL* (Left) on day 14 of co-culturing hiPSC-MSCs and -macrophages in a monolayer with or without osteogenic supplements. Data are averages ± SD (n = 3). **p < 0.01 indicate significant difference between osteogenic supplements (−) and (+) in the hiPSC-MSC/-macrophage; ^$^p < 0.05, ^$$$^p < 0.001 indicate significant difference between hiPSC-MSC/-macrophage and hiPSC-MSC without osteogenic supplements. (**c**) Schema for protocol and timeline. hiPSC-MSCs were seeded and allowed to attach to the HA-based composite scaffolds in MSC growth medium for 1 day. Medium containing osteogenic supplements was used to commit these cells to the OB lineage for 7 days, after which it was seeded with hiPSC-macrophages and incubated without osteogenic supplements, containing M-CSF and RANKL, to differentiate into both OB and OC lineages for 10 days. hiPSC-derived multicellular bone constructs were either grown *in vitro* or implanted subcutaneously into athymic nude mice for 8 weeks.

Osteogenic supplements dexamethasone and β-glycerophosphate inhibit monocyte differentiation into OC formation and their related function^18^,^19^. Thus, we first focused on identifying an optimal culture regimen to ensure development of OC by inducing the differentiation of hiPSC-MSCs in medium with osteogenic supplements dexamethasone, β-glycerophosphate, and 1α,25-dihydroxyvitamin D_3_ followed by co-culturing with hiPSC-macrophages either with (+) or without (−) osteogenic supplements in a monolayer. Co-culture without osteogenic supplements exhibited large multinucleated OCs with diameters of 50–100 *μ*m embedded in OBs (Fig. 1a) in contrast to co-culture with osteogenic supplements. In the absence of osteogenic supplements, there were significant increases in the expression of OC-related genes nuclear factor-activated T cells c1 (*NFATC1*), calcitonin receptor (*CTR*), and cathepsin K (*CATK*), as well as *RANKL*, which is expressed by OBs, compared to co-culture in osteogenic supplements (Fig. 1b). OB-related gene expression of *OCN* was higher for both hiPSC-derived cultures under the osteogenic supplements, which showed a typical high value due to the addition of osteogenic supplements. As a reference, the expression levels of all OC-related markers were not detected in the hiPSC-MSC mono-culture with and without osteogenic supplements. Thus, OCs could be differentiated from hiPSC-macrophages in osteogenic supplement–free medium when co-culturing with hiPSC-derived OBs.

The established monolayer co-culture method was then adapted for 3D tissue-engineered model of bone to evaluate whether crosstalk between these cells improves bone formation over existing mono-culture approaches (Fig. 1c). We first seeded hiPSC-MSCs onto the scaffolds with varying amounts of HA (HA-0, -1, and -5: poly(lactic-*co*-glycolic acid)/poly(L-lactic acid) (PLGA/PLLA) scaffold with 0%, 1%, and 5% w/v HA, respectively). The cells were allowed to attach on the surface of the scaffolds and adapt to the new environment under MSC growth medium supplemented with basic fibroblast growth factor (b-FGF) for 2 days. Subsequently, the hiPSC-MSC-seeded scaffolds were differentiated into OBs by cultivation with osteogenic supplements for 7 days. The scaffold was then seeded with hiPSC-macrophages and incubated for an additional 5 days, without osteogenic supplements, but with M-CSF followed by RANKL and M-CSF for another 5 days. These cell-seeded scaffolds were either grown *in vitro* to develop bone tissue for analysis or implanted subcutaneously into athymic nude mice (an ectopic bone regeneration model).

### Adding hiPSC-macrophages to hiPSC-MSCs accelerates *in vitro* bone tissue formation

**Figure 2:**
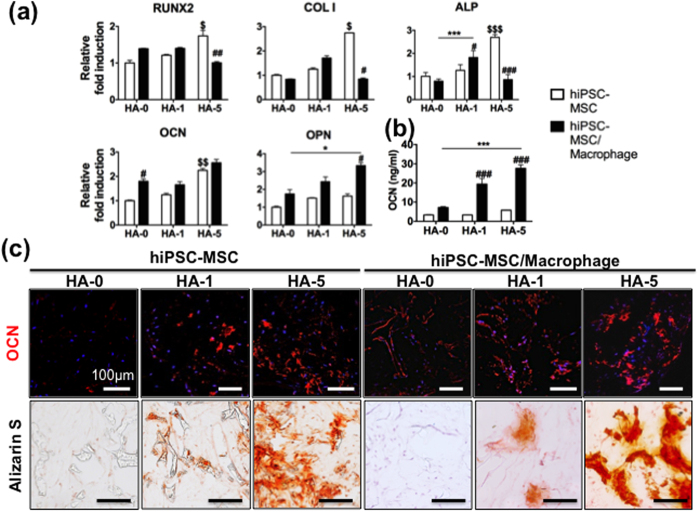
hiPSC-macrophage addition to hiPSC-MSC culture accelerates *in vitro* bone tissue formation. Bone tissue development from hiPSC-MSCs was compared with hiPSC-MSCs/-macrophages on PLGA/PLLA scaffolds or HA composite PLGA/PLLA scaffolds for 3 weeks. (**a**) Quantitative real-time PCR of gene expression for osteogenic markers *RUNX2, COLI, ALP*, *OCN,* and *OPN*. (**b**) OCN released into culture medium, indicative of bone maturation. Data in (**a**,**b**) are averages ± SD (n = 3). *p < 0.05, ***p < 0.001 indicate significant difference between HA-0 and the other doses of HA in the hiPSC-MSC/-macrophage; ^#^p < 0.05, ^##^p < 0.01, and ^###^p < 0.001 indicate significant difference between hiPSC-MSC/-macrophage and hiPSC-MSC in the same dose of HA; ^$$^p < 0.01, ^$$$^p < 0.001 indicate significant difference between HA-0 and the other doses of HA in hiPSC-MSC. (**c**) Immunofluorescent staining for OCN and Alizarin Red S staining of hiPSC-MSC and hiPSC-MSC/-macrophage in the various concentrations of HA-based scaffolds. HA-0: PLGA/PLLA scaffold, HA-1: PLGA/PLLA scaffold with 1% w/v HA; HA-5: PLGA/PLLA scaffold with 5% w/v HA. Scale bars, 100 *μ*m.

In the engineered 3D constructs with only hiPSC-MSCs, after 28 days there was a noticeable increase in expression of OB-related genes, including *RUNX2*, *COLI*, *ALP*, *OCN* in HA-5 compared with no HA (Fig. 2a). When hiPSC-MSCs were co-cultured with hiPSC-macrophages in HA-5, there was a shift in osteogenic gene expression: increases in expression of osteopontin (*OPN)* and release of OCN as later stage of osteogenic markers^20^ (Fig. 2a,b), with a concomitant decrease in early osteogenic markers *COLI* and *ALP* as well as *RUNX2* transcriptional expression. Co-cultured cells on HA-5 composite scaffolds produced more maturation of bone-specific matrix and mineralization than HA-1 and HA-0 scaffolds (Fig. 2c).

**Figure 3:**
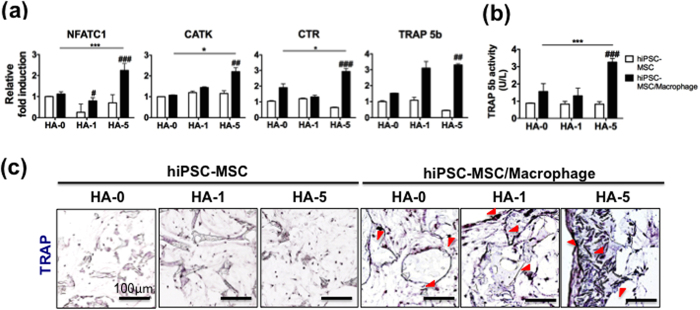
hiPSC-macrophages differentiated into OCs in HA-based co-culture bone constructs *in vitro*. Osteoclastogenic potential of hiPSC-macrophages was characterized *in vitro* in hiPSC-MSC/-macrophage and hiPSC-MSC on PLGA/PLLA scaffolds or HA composite PLGA/PLLA scaffolds for 3 weeks. (**a**) Quantitative real-time PCR of gene expression for OC markers *NFATC1*, *CATK, CTR,* and *TRAP 5b*. (**b**) TRAP5b released into culture medium, indicative of OC cell number. Data in (**a,b**) are averages ± SD (n = 3). *p < 0.05, ***p < 0.001 indicate significant difference between HA-0 and the other doses of HA in hiPSC-MSC/-macrophage; ^#^p < 0.05, ^##^p < 0.01, and ^###^p < 0.001 indicate significant difference between hiPSC-MSC/-macrophage and hiPSC-MSC in the same dose of HA. (**c**) Histological analysis of TRAP-positive OCs (red arrowhead) in hiPSC-MSC and hiPSC-MSC/-macrophage in various HA-based scaffolds. HA-0: PLGA/PLLA scaffold, HA-1: PLGA/PLLA scaffold with 1% w/v HA; HA-5: PLGA/PLLA scaffold with 5% w/v HA. Scale bars, 100 *μ*m.

In HA-5 3D composite scaffolds with co-culture, hiPSC-macrophages appeared well-differentiated into OCs, as demonstrated by higher expression of *NFATC1*, *CATK*, *CTR,* and *TRAP 5b* (Fig. 3a) and TRAP 5b enzyme activity than HA-0 and -1 composite scaffolds (Fig. 3b). TRAP-positive OCs were observed at the construct edges or scaffold surfaces in the HA-containing co-culture scaffolds (Fig. 3c, red arrowhead). As a reference, the expression levels of all OC-specific markers and TRAP activity were not detected in the hiPSC-MSC mono-culture under the same conditions, indicating that OC differentiation did not occur. Overall, the accelerated tissue development in the HA-based co-culture constructs suggests the important role of a dynamic interplay between OCs (TRAP activity) and OBs (OCN presence) in bone maturation.

### hiPSC-MSC/-macrophage co-culture induces mature bone-like tissue formation *in vivo*

**Figure 4:**
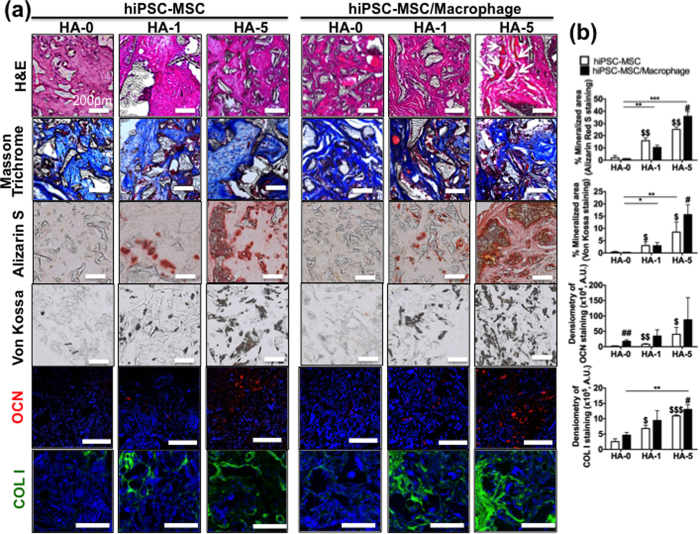
hiPSC-MSC/-macrophage co-culture induces mature bone-like tissue formation *in vivo* in mice. hiPSC-MSCs and hiPSC-MSC/-macrophages on PLGA/PLLA scaffolds or HA-composite PLGA/PLLA scaffolds were implanted subcutaneously for 8 weeks in the athymic nude mice. (**a**) Histology revealed deposition of osseous tissue (H&E; arrow) and collagen (Masson’s Trichrome: blue), calcium and phosphate deposits (Alizarin Red S and Von Kossa), and OCN expression. (**b**) Quantification of OCN and COLI intensity and calcified area based on Alizarin Red S and Von Kossa staining. Data are averages ± SD (n = 3). *p < 0.05, **p < 0.01, ***p < 0.001 indicate significant difference between HA-0 and the other doses of HA in hiPSC-MSC/-macrophage; ^#^p < 0.05, ^##^p < 0.01 indicate significant difference between hiPSC-MSC/-macrophage and hiPSC-MSC in the same dose of HA; ^$^p < 0.05, ^$$^p < 0.01, ^$$$^p < 0.001 indicate significant difference between HA-0 and the other doses of HA in hiPSC-MSC. HA-0: PLGA/PLLA scaffold, HA-1: PLGA/PLLA scaffold with 1% w/v HA; HA-5: PLGA/PLLA scaffold with 5% w/v HA. Scale bars, 200 *μ*m.

To determine whether hiPSC-MSCs co-cultured with hiPSC-macrophages within the 3D scaffold could maintain stable osteoblast and osteoclast lineage commitments and constitute functional bone tissues *in vivo*, we implanted constructs subcutaneously in athymic nude mice. Eight weeks after implantation, explanted scaffolds (lacking HA) from mono-cultured hiPSC-MSCs were filled with fibrous connective tissue, but did not have bone matrix deposition. However, hiPSC-MSCs on HA-5 composite scaffolds produced copious osseous tissue and high levels of OCN, COLI, and calcium deposition, resulting in faster bone formation than HA-0 and -1 composite scaffolds (Fig. 4a). In agreement with our *in vitro* finding, the co-culture constructs formed more mature bone with lamellar structure (H&E), organized collagen fibers (Masson’s trichrome), more OCN and COLI production, and osteocytes embedded in mineralized extracellular matrix (Alizarin Red S and Von Kossa) in HA-5 composite scaffolds compared with the hiPSC-MSC mono-culture (Fig. 4a). Semi-quantitative analysis of these markers of bone formation further confirmed that hiPSC-MSC/-macrophage on HA-5 composite scaffolds formed large areas of mineralized bone in the form of calcium and phosphate deposits with concomitant increases in OCN and COLI production, compared with mono-cultured hiPSC-MSCs (Fig. 4b). No evidence of teratoma around the site of implant was observed in any of the scaffolds at 8 weeks. We also confirmed the human origin of engineered bone tissues for all scaffolds by staining for human lamin A+C (Fig. S3).

We then analyzed functional behavior of OCs in the engineered bone constructs from hiPSC-MSC/-macrophage co-cultures *in vivo*. Histological examination revealed that TRAP-positive and CATK-expressing OCs in co-culture were more homogeneous and abundant, in a HA-dose dependent manner, compared with mono-cultured hiPSC-MSCs (Fig. S4). Mono-cultured hiPSC-MSCs showed similar, but much lower, production of the TRAP resorbing enzyme at the construct edges or surfaces of scaffolds than co-cultured hiPSC-MSC/-macrophage. These data point toward active bone resorption by osteoclastogenic hiPSC-macrophages in HA-containing scaffolds, as well as bone formation and functional maturation by hiPSC-derived OBs.

### Coordinated hiPSC-derived OB and OC activity leads to engineered bone-like tissues

**Figure 5:**
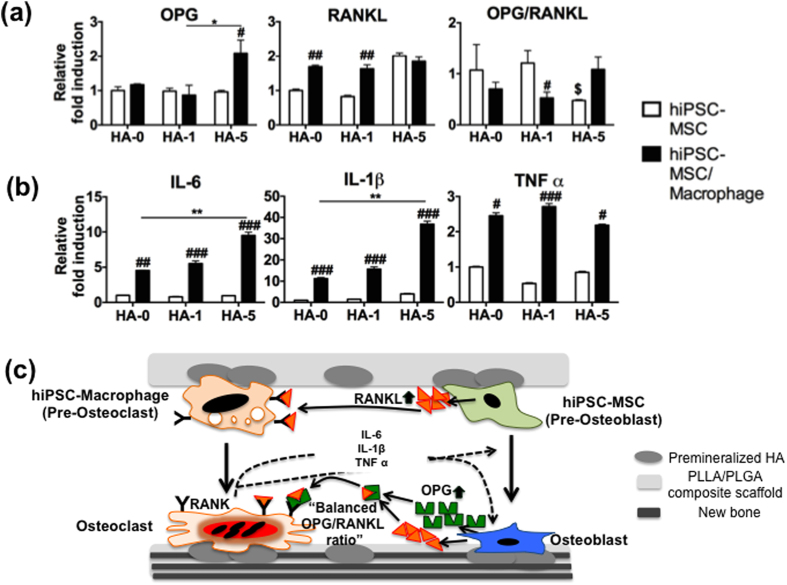
Well-orchestrated coupling of hiPSC-derived OBs and OCs leads to bone-like engineered tissues. Bone tissue was created from hiPSC-MSC and hiPSC-MSC/-macrophage on PLGA/PLLA scaffolds or HA composite PLGA/PLLA scaffolds for 3 weeks. (**a**) Expression of coupled factors *OPG* and *RANKL*, and their ratio. (**b**) Expression of cytokines *IL-6, IL-1β,* and *TNFα*. Quantitative real-time PCR data in (**a**,**b**) are averages ± SD (n = 3). *p < 0.05, **p < 0.01 indicate significant difference between HA-0 and the other doses of HA in hiPSC-MSC/-macrophage; ^#^p < 0.05, ^##^p < 0.01, and ^###^p < 0.001 indicate significant difference between hiPSC-MSC/-macrophage and hiPSC-MSC in the same dose of HA; ^$^p < 0.05 indicates significant difference between HA-0 and the other doses of HA in hiPSC-MSC. (**c**) Schematic for possible coupling mechanisms among hiPSC-derived OBs, OCs, and their progenitors under interaction with HA microenvironment for bone tissue development. HA-0: PLGA/PLLA scaffold, HA-1: PLGA/PLLA scaffold with 1% w/v HA; HA-5: PLGA/PLLA scaffold with 5% w/v HA.

We next examined the molecular details of the pathways that accelerated bone-like tissue formation in our HA scaffolds. OPG/RANKL are key molecular coordinators expressed by OBs that regulate bone resorptive activity of OCs and are responsible for the homeostatic mechanism of bone remodeling^21^. RANKL stimulates OC precursors to commit to the OC phenotype by binding to its receptor RANK, which is present on precursor and mature OCs. OPG is a suppressor of osteoclastic bone degradation by competing with RANK for binding of RANKL. In the engineered constructs from hiPSC-MSCs treated *in vitro* with osteogenic supplements for 28 days, the expression of *OPG* remained unchanged in varying HA concentrations while *RANKL* expression increased two fold in HA-5 composite scaffolds (Fig. 5a). However, the constructs with hiPSC-MSCs/-macrophages co-cultured showed a significant increase in both *OPG* and *RANKL* expression in HA-5 composite scaffolds. These data suggest that the high dose of HA prevents unintended, excessive hiPSC-macrophage osteoclastogenesis by increasing OPG production, which blocks the actions of RANKL in the co-culture. As a consequence, the ratio of *OPG/RANKL*, which determines the extent of OC activity^22^, was close to 1 in HA-5 with hiPSC-MSC/-macrophage co-culture, further supporting balanced bone formation and resorption processes.

We also assessed several inflammatory molecules, expressed by OCs and their precursors, that bound directly to OB precursors to enhance the differentiation and function of OBs^23^. Co-culture systems with a higher percentage of HA resulted in a dose-dependent up-regulation of *IL-6* and *IL-1β* expression with no significant change in *TNFα* in all scaffolds (Fig. 5b). However, the expression of *IL-6*, *IL-1β*, and *TNFα* did not change markedly in all scaffolds when only hiPSC-MSCs were cultured under osteogenic conditions. Taken together, our findings suggest that co-cultured cells on the HA-based constructs induce coupling signals coordinating OB and OC activity, resulting in enhanced bone-like tissue formation.

## Discussion

The field of tissue engineering is increasingly focused on harnessing hiPSCs and their derivatives with the combined use of biomaterials to build or regrow patient-specific tissues, such as bone. hiPSCs developed from a patient’s own somatic cells have an indefinite potential to differentiate into all cell types residing in the human bone—osteoblast, osteocyte, and immune cells, such as monocytes, macrophages, and osteoclasts^6^,^13^. However, current bone tissue engineering approaches, which primarily rely on the cultivation of MSCs as bone-forming osteoblast precursors, do not produce functional bone substitutes in a way akin to the physiological bone regeneration process. In the body, bone cells (mainly osteoblasts and osteoclasts) coordinate and balance their respective anabolic and catabolic processes to maintain functional equilibrium during the extracellular matrix remodeling process^24^. Thus, an attempt to recreate the crosstalk between these two cell types, using hiPSCs and biomaterial platforms, could produce functional and sophisticated bone substitutes.

Recent studies have similarly evaluated using hiPSCs for bone regeneration^7^,^10^,^11^,^25^,^26^,^27^. However, our data highlighted the need for bone extracellular matrix remodeling that is modulating through immune cells being required for maturation of bone by including hiPSC-derived OCs in addition to OBs. We hypothesized that the formation of new bone *in vivo* requires a balance between the anabolic activity of osteoblasts and the catabolic function of osteoclasts. We report a novel 3D human bone model that was engineered by co-culturing hiPSC-derived MSCs and macrophages on HA-coated polymer scaffolds—cells and materials that together mimic both function and structure of native human bone^10^. In our *in vitro* study, the composite scaffolds with higher percentage of HA not only induce the osteoclastic differentiation of hiPSC-Macrophage (by *NFATC1*, *CATK*, *CTR*, and *TRAP5b*) but also stronger osteogenic activity of hiPSC-MSCs compared to low HA or PLLA/PLGA alone. Notably, co-cultured cells with HA-based composites showed the expression level of *OCN* and *OPN* increased while early osteogenic markers including *COL1* and *ALP* decreased compared with mono-cultured hiPSC-MSC, indicating differentiation into mature OBs. Decreased *RUNX2* observed in co-cultured hiPSC-MSC/-macrophage on high-HA composites could be due to the temporal changes in its mRNA expression during osteogenesis^28^.

Our ectopic bone formation model demonstrated that unlike the composite scaffolds containing low HA or without HA, where fibrous connective tissues are found throughout the constructs, in high-concentration HA scaffolds co-cultured hiPSC-MSC/-macrophage formed mature bone with lamellar collagen fibers and increased nonmineralized (by OCN and COLI) and mineralized bone matrix deposition. The underlying mechanisms appear to involve the balanced OPG/RANKL ratio that mediate the coordinated activity between actions of bone-building osteoblasts and bone-resorbing osteoclasts critical for bone remodeling^21^. Like our previous study^15^, this could be attributed that special biochemical and biophysical cues created by the addition of HA particles preferentially direct the differentiation of hiPSC-MSCs/-macrophages into OBs and OCs, thereby allowing for functional engraftment of hiPSC-derived cells into engineered bone tissues.

Osteoblasts control the formation and activity of osteoclasts and, therefore, the resorption of bone through coupling mechanisms such as RANK, RANKL, and OPG^29^,^30^. Conversely, as progenitors for OCs, macrophages and their monocyte precursors play a pivotal, non-immunological regulatory role in bone formation, regeneration, and homeostasis *in vivo*^23^,^31^. Indeed, bone-specific macrophages named osteal macrophages have been identified recently in human bone and secrete inflammatory molecules to facilitate bone formation and remodeling^32^,^33^. There are also clinical reports that the inhibition of OC-mediated bone resorption by bisphosphonate administration in patients with osteoporosis developed osteonecrosis of the jaw^34^, implying an important role of immune cells in supporting the bone repair and regeneration process. It is clear from our *in vitro* and *in vivo* studies that macrophages and differentiated OCs in combination with OBs are necessary for engineering mature bone-like tissues.

We also verified that bone-specific signaling pathways were reproduced in our engineered scaffold co-cultures. IL-6, for instance, is a potent cytokine secreted by osteoclasts and their precursors, which facilitates calvarial bone formation in mice^35^, and IL-1β induces MSC differentiation into osteoblasts^36^ and is required for tissue ingrowth and bone formation in rabbits^37^. TNFα plays an essential role in commitment to the osteoclastic lineage and activation of mature OCs by precisely tuning IL-6, RANKL, M-CSF, and OPG^38^. Here, we found that the induction of IL-6, IL-1β, and TNFα—expressed by hiPSC–derived OCs and their precursor macrophages in our HA-based constructs—worked together to stimulate OB differentiation and bone formation, indicating the necessity of crosstalk between these bone cell types (Fig. 5c). Furthermore, promotion of RANKL production by OBs induced differentiation of hiPSC-macrophages into OCs. Interestingly, OPG synthesis was stimulated by cellular interaction with the HA microenvironment, and the presence of OPG counteracted the action of RANKL on OCs. Thus, the balance of bone formation versus resorption was improved by pairing co-cultured cells with HA-based composites. These findings suggest that local cues provided by the HA niche can guide intercellular signaling between hiPSC-MSCs and -macrophages to more accurately mimic bone physiology. Similar to wound healing^39^, it is clear that cytokines and regulatory molecules secreted by immune cells (macrophages and OCs) can tightly regulate extracellular matrix remodeling required during development and maturation of bone.

Our 3D bone tissue model engineered from hiPSCs not only elicited controlled differentiation of two bone cell precursors, but also demonstrated preliminarily functional regeneration of human bone-like tissue *in vivo*. Further investigation in orthotopic implantation models will be required to validate the functionality, especially for vascularization, which leads to better bone formation^40^, and to confirm safety of bone grafts engineered from hiPSCs in the long run. From a translational standpoint, our 3D tissue-engineered bone using patient-specific iPSCs could capture the functional interplay between osteoclastic and osteoblastic activities and thus, it could be used in understanding of bone cell phenotypes in the pathology of bone disease and in the testing of drugs regulating bone homeostasis.

## Materials and Methods

### EB formation from hiPSC

The MJ hiPSC used in this study were derived from a healthy individual and have been previously described^41^. For embryoid body (EB) formation, hiPSC were detached from plates and transferred to six-well ultra-low-attachment plates. To differentiate three germ layers *in vitro*, EBs were maintained in EB culture medium for 15–20 days with half of the medium changed every other day. EB medium consisted of DMEM-F12 (Invitrogen), 20% (v/v) Knockout Serum Replacement (Invitrogen), 1 mM L-glutamine, 0.1 mM β-mercaptoethanol (β-ME), and 1X nonessential amino acids (NEAA)^41^.

### Differentiation (adipogenic, chondrogenic, and osteogenic) and characterization of MSCs from hiPSCs

To generate mesenchymal progenitors, the EBs were transferred onto tissue culture plates coated with gelatin (0.1% w/v) and cultured for 10 additional days until the outgrowth from the EBs occurred in MSC growth medium consisting of DMEM (Invitrogen) supplemented with 10% FBS (Hyclone), 1% Glutamax (Invitrogen), 100 U/ml penicillin-streptomycin (Invitrogen), and 8 ng/mL basic fibroblast growth factor (bFGF) (PeproTech). Migrating cells isolated from EBs were subcultured at an initial cell density of 2 × 10^4^ cells/cm^2^ and subsequently expanded as MSCs under the same conditions (3–5 passages used for all experiments).

For adipogenic differentiation, we cultured the hiPSC-derived MSCs at 1 × 10^4^ cells/cm^2^ for three weeks in adipogenic media consisting of DMEM, 10% FBS, 100 U/ml penicillin-streptomycin, 1 *μ*M dexamethasone, 100 *μ*M indomethacin, 500 *μ*M 3-isobutyl-1-methtlxanthine (IBMX), and 10 *μ*g/ml Insulin (all from Sigma). To evaluate adipogenesis, lipid droplets were stained with 30 mg/ml oil red O (Sigma-Aldrich) in 60% isopropanol after fixation of cells with 10% formaldehyde.

Chondrogenic differentiation of hiPSC-MSCs was evaluated in 3D micromass cultures. Cells (5 × 10^4^) were seeded in the 96-well MicroWell™ round bottom plate (Thermo Fisher Scientific) and the 3D micromass was formed in the bottom by centrifuging at 2000 rpm for 5 min. The micromass was maintained at 37 °C with 5% CO_2_ in the chondrogenic media consisting of DMEM, 10% FBS, 100 U/ml penicillin-streptomycin, 1% ITS premix (BD Bioscience), 100 mM sodium pyruvate (Life Technologies), 40 mg/ml L-proline (Sigma), 50 mM ascorbic acid-2-phosphate, and 10 ng/mL of transforming growth factor-β1 (TGF-β1) (PeproTech). To evaluate chondrogenesis, the micromass was harvested after 4 weeks, fixed in 4% paraformaldehyde overnight, dehydrated in increasing concentrations of ethanol, embedded with paraffin, and sectioned at 5 *μ*m. The amount of proteoglycans was evaluated by aqueous Safranin-O (0.1%) using standard procedures.

For osteogenic differentiation, 1 × 10^4^ hiPSC-derived MSCs were cultured for 3 weeks in osteogenic media composed of DMEM, 10% FBS, 10% FBS, 100 U/ml penicillin-streptomycin, 100 nM dexamethasone, 10 mM β-glycerophosphate, and 0.1 mg/mL ascorbic acid-2-phosphate (all from Sigma). To assess osteogenic potential (calcium deposition), cells were fixed with 10% formaldehyde, rinsed with distilled water, and stained with Alizarin red [Sigma; 0.5% (v/v) in distilled water (pH 4.2)] for 10 min. For ALP staining, cells were rinsed with Tyrode’s balanced salt solution, fixed with citrate-buffered acetone, and incubated in a mixture of fast violet B salt (Sigma) and naphthol AS-MX phosphate (3-(Phosphonooxy)-N-(2,4-xylyl)naphthalene-2-carboxamide) solution (Sigma).

### Differentiation and characterization of osteoclastogenic monocytes/macrophages from hiPSCs

Directed differentiation of hiPSC to monocytes/macrophages was carried out as previously described^17^,^42^. Briefly, for EB formation, hiPSCs were detached from plate and feeder cells by treatment with 0.2% dispase for 4 min and were collected by scraping. The hiPSCs were transferred into 6-well, ultra-low-attachment plates (Costar) in EB culture medium and were cultured for 4 d at 37 °C. For monocyte differentiation, 4–10 large EBs were transferred into gelatin-coated six-well plates containing monocyte differentiation media comprised of DMEM, 10% FBS, 50 ng/mL human macrophage colony-stimulating factor (hM-CSF) (PeproTech), 25 ng/mL human IL-3 (PeproTech), 1 mM L-glutamine, 1× NEAA, and 0.1 mM β-ME. Continuous monocyte production started within 15–20 d and monocytes were harvested every 4–5 d for 2 months^41^.

For macrophage differentiation, monocytes harvested from EB factories were resuspended in macrophage differentiation medium comprised of RPMI, 10% FBS, 100 ng/mL hM-CSF, glutamine, and 100 U/ml penicillin-streptomycin and plated at a density of 3–5 × 10^4^ cells in 24 well plates or Corning^R^ osteo assay surface 96-well plates, changing the media once in two days. For osteoclast differentiation, after 3 days of seeding, we added 100 ng/ml recombinant human soluble RANK ligand in macrophage differentiation medium for 3 weeks. Osteoclastogenic potential was assessed by TRAP and CATK-positive osteoclastic cells. Resorbed calcium phosphate was visualized by Von Kossa staining after the removal of all cells on osteo assay surface 96-well plates. Briefly, the plates were stained with 5% silver nitrate solution under ultraviolet light for 15 min, washed three times with distilled water, and incubated with 5% sodium thiosulfate for 5 min at room temperature under regular light to quench unreacted silver. Then images were acquired by using a light microscope. In addition, iPSC-osteoclasts were stained for cathepsin K using mouse anti-cathepsin K (Millipore) and for F-actin using Alexa fluor 568 phalloidin (Life technologies).

### Polymer scaffold preparation

The HA composite scaffolds composed of a 1:1 ratio of poly(lactide-*co*-glycolide) (PLGA) (Sigma, Mw ≈ 30 kDa) and poly(L-lactic acid) (PLLA) (Sigma, Mw ≈ 55 kDa) were fabricated by salt-leaching method, as described previously^15^. Briefly, PLGA and PLLA (1:1) were dissolved in chloroform to yield a solution of 5% (w/v) polymer with or without HA particles (Sigma, 1% or 5% w/v). Sponges (1 × 1 × 0.5 cm^3^) were sterilized overnight in 75% (v/v) ethyl alcohol overnight, washed three times with PBS, and coated with fibronectin (10 ng/ml) for 3 h before cell seeding.

### Flow cytometry

hiPSC-MSCs, -monocytes, and -macrophages were harvested and resuspended to 3 × 10^5^ cells in 50 *μ*l of PBS containing 0.1% BSA. Cells were separately labeled with phycoerythrin (PE)-conjugated mouse anti-human antibody CD44 (Millipore), CD68 and CD73 (Pharmingen), FITC-conjugated mouse anti-human antibody CD105, CD45 (Pharmingen), and CD115 (Millipore), and APC-conjugated mouse anti-human antibody CD14 and CD68 (Pharmingen) on ice for 30 min. An isotype-matched mAb was used as a control (Becton Dickinson). Data were analyzed with the BD Accuri^TM^ C6 flow cytometer and FlowJo X 10.0 software (Becton Dickinson).

### RNA extraction and real-time RT-PCR

Total RNA was extracted from cells, micromass, and HA composite scaffolds using Trizol reagent (Life Technologies) and cDNA was synthesized by using Superscript(R) II reverse transcriptase following the manufacturer’s protocol (Invitrogen, Carlsbad, CA). Real-time PCRs were performed using StepOnePlus(R) Real Time PCR System (Applied Biosystems, Carlsbad, CA) with SYBR Green PCR Master Mix (Life Technologies). The relative expression of each target was calculated using the ΔΔCT method and β-actin were used as endogenous references. All expression levels of samples were normalized to controls. The PCR primers used for RT-PCR are listed in Table S1. Expression of all markers was normalised to the expression of the housekeeping gene β-actin.

### Osteocalcin and TRAP 5b ELISA assay

The osteocalcin ELISA assay was performed with the MicroVue Osteocalcin EIA Kits (Cat 8002; Quidel) following the manufacturer’s instructions. The TRAP ELISA assay was performed with the BoneTRAP (TRACP 5b human) ELISA (Cat SB-TR201R; ids) following the manufacturer’s instructions. For assaying the osteocalcin and TRAP released from HA composite scaffolds, a serum-free medium was used 48 h before protein harvest. Optical density was measured at 405 nm using the microplate reader (Biotek). Samples were analyzed in triplicate for secreted soluble osteocalcin and TRAP using quadratic curve fit.

### *In vivo* subcutaneous transplantation

hiPSC-derived MSCs were expanded (passage 4), seeded (1.5 × 10^6^) onto the polymer sponges (1 × 1 × 0.5 cm^3^), and cultured under MSC growth medium for 1 day. After treatment with an osteogenic-supplement dexamethasone and β-glycerophosphate and 10 nM 1α,25 dihydroxyvitamin D3 (Sigma) for 7 days, hiPSC-MSC/scaffolds were seeded with hiPSC-macrophages (passage 0; 1.5 × 10^6^) and incubated for 5 days without osteogenic-supplements but containing 50 ng/ml M-CSF followed by co-culture in the presence of 100 ng/ml RANKL and 100 ng/ml M-CSF for another 5 days. The cell-seeded scaffolds were implanted subcutaneously into the dorsal region of 6-week-old athymic female nude mice (Charles River Laboratories; n = 18) and the skin was closed with a nylon suture. Constructs were harvested after 8 weeks and processed for histology. All procedures were performed under a pre-established protocol (The Johns Hopkins University Animal Care and Use Committee approved the animal procedures, protocol number MO14M126).

### Histological analysis of explanted scaffolds

The tissue constructs were fixed in 10% formalin, embedded in optimum cutting temperature (OCT) compound (Tissue-Tek, Sakura Finetek), and cryosectioned (8 *μ*m thick). The cryosections were immediately stained with hematoxylin and eosin, Masson’s Trichrome, Alizarin Red S, Von Kossa, or TRAP or stored at −80 °C until use. Images of Alizarin Red S and Von Kossa staining were quantified using ImageJ (NIH). Briefly, a minimal intensity threshold was used to eliminate the background and then the stained Alizarin Red S and Von Kossa were measured as image % area coverage.

For immunohistochemistry, the sections were blocked with 20% normal goat serum in 2% BSA for 30 min, and incubated with rabbit polyclonal antibodies against osteocalcin (Millipore) (1:100 dilution), type I collagen (Abcam) (1:100 dilution), and cathepsin K (Biovision) (15 *μ*g/ml dilution) at 4 °C overnight. The sections were then incubated with Alexa Fluor 594 goat anti-rabbit secondary antibody H+L (Life Technologies) (1:300 dilution) or FITC-conjugated affinipure goat anti-rabbit secondary antibody H+L (Jackson Immunoresearch) (1:50 dilution) for 1 h and counterstained with DAPI (Chemicon) for 10 min. Human-specific nuclei staining was also performed with mouse IgG1 human lamin A+C (Abcam) (1:100 dilution) and goat anti-mouse Alexa fluor 488 (Life technology) (1:400 dilution). All images were collected with a Zeiss LSM Metal Confocal microscope. The intensity of osteocalcin and type II collagen was quantified as the integrated density subtracted from the mean fluorescence of background using the ImageJ software (NIH).

### Statistical analysis

All analyses were performed in triplicate samples for n = 3 at least. Real time RT-PCR was also performed on triplicate samples (n = 3) with triplicate readings. Data are expressed as average +/− standard deviation and the statistical significance (p value) was determined by One-way analysis of variance (ANOVA) with the Turkey post hoc test using GraphPad Prism 5.

## Additional Information

**How to cite this article**: Jeon, O. H. *et al.* Human iPSC-derived osteoblasts and osteoclasts together promote bone regeneration in 3D biomaterials. *Sci. Rep.* **6**, 26761; doi: 10.1038/srep26761 (2016).

## Supplementary Material

Supplementary Information
