# Supplementary material for: Human iPSC-derived osteoblasts and osteoclasts together promote bone regeneration in 3D biomaterials
Source: Sci Rep. 2016 May 26;6:26761. doi: 10.1038/srep26761 (PMC4881234; doi:10.1038/srep26761)
Supplement: Supplementary Information [file srep26761-s1.doc]

**Title: Human iPSC-derived osteoblasts and osteoclasts together promote bone regeneration in 3D biomaterials**

Ok Hee Jeon, Leelamma Panicker, Qiaozhi Lu, Jeremy Chae, Ricardo Feldman, Jennifer Elisseeff

**Supplemental Figures**

**
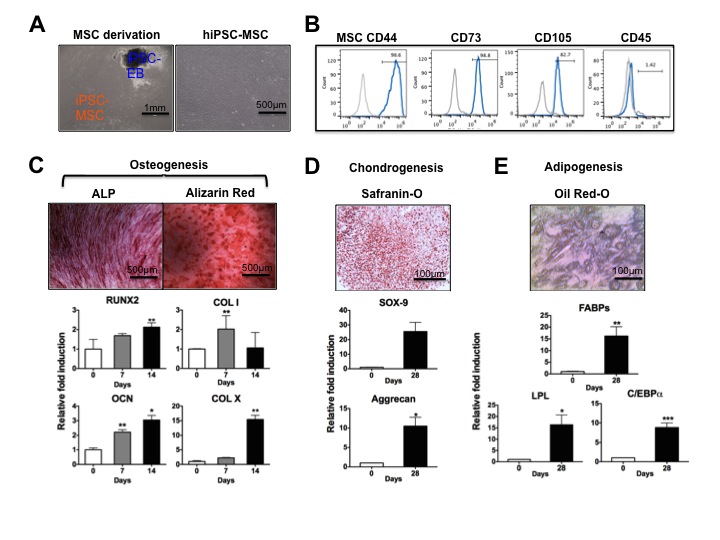
**

**Supplementary Fig. 1 Isolation and characterization of hiPSC derived-MSC.** (A) Light microscopy of hiPSC-MSCs migrating out of the embryoid body (EBs) for a week (Left) were harvested and expanded in monolayer culture with MSC growth medium (Right). (B) Flow cytometry analysis of hiPSC-MSCs indicates that these cells express MSC surface markers (at passage 3). Histograms show the percentage of cells stained with antibodies to specific markers (Blue) and isotype controls (Grey). *In vitro* differentiation potential of hiPSC-MSCs towards: (C) an osteogenic lineage (ALP and Alizarin red staining and gene expression of *RUNX2, COL I, COL X,* and *OCN* after 7 and 14 days in monolayer cultures); (D) a chondrogenic lineage (proteoglycan production confirmed by Safranin-O staining and gene expression of *SOX-9* and *Aggrecan*); and (E) an adipogenic lineage (Oil Red-O staining and gene expression of *FABPs, LPL*, and *C/EBPα*). Data in (C-E) are averages ± SD (n=3). ^*^p < 0.05, ^**^p < 0.01, and ^***^p < 0.001 versus day 0.

**
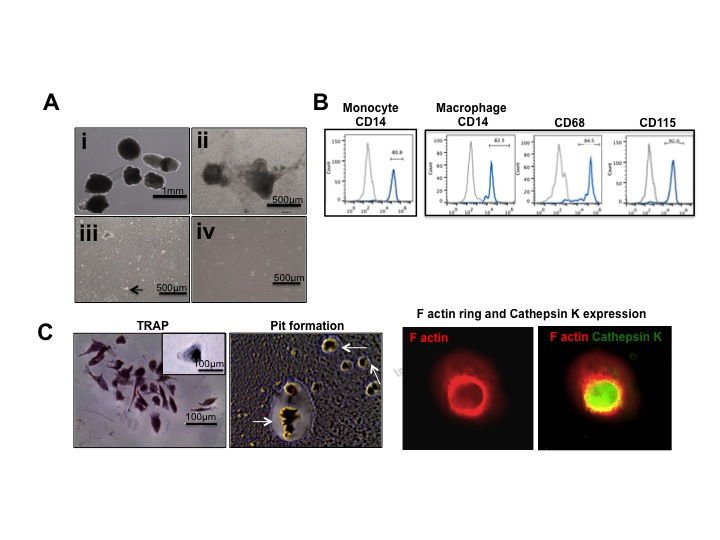
Supplementary Fig. 2 Isolation and characterization of hiPSC derived-macrophage and their potential of osteoclastogenesis.** (A) A stepwise derivation strategy for differentiation of macrophage from hiPSCs. (i) Ten-day-old embryoid body (EBs) in suspension culture, (ii) flattened EBs that were ready to produce monocytes after 17 d with monocyte differentiation medium, (iii) EB factories producing monocytes (Floating round monocyte-like in the culture medium, arrow), and (iv) monocytes differentiated into macrophages after plating onto adherent tissue culture plates for 5 days with macrophage differentiation medium supplemented with M-CSF and IL-3. (B) Flow cytometry analysis of hiPSC-monocytes and macrophages. Histograms show the percentage of cells stained with antibodies to specific markers (Blue) and isotype controls (Grey). (C) Differentiation of osteoclast from hiPSCs shown by TRAP-positive osteoclastic cells (left), resorption pits created by functional hiPSC-derived OCs (arrow; Middle), and colocalization of cathepsin K (green) with F-actin ring (red; Right).

**
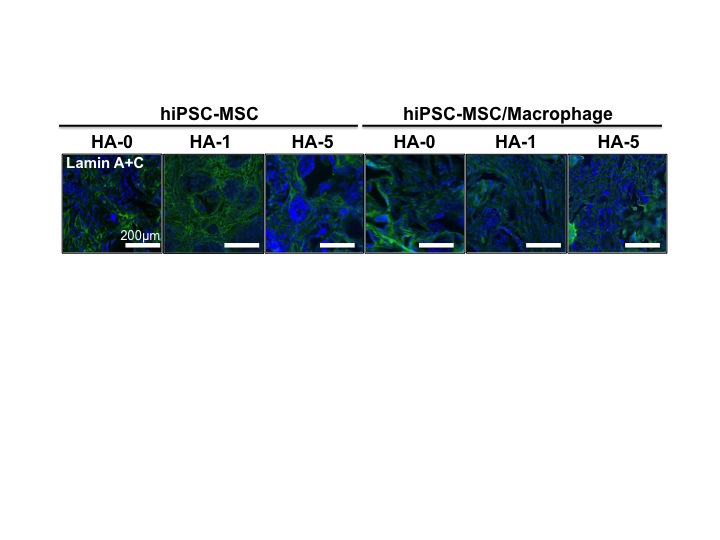
Supplementary Fig. 3 Anti-human nuclei staining of Lamin A+C confirming human origin of 3D engineered bone tissue.** Human origin of the cells was confirmed by anti-human nuclei staining of Lamin A+C both in the engineered bone tissue for all hiPSC-MSC (Left) and hiPSC-MSC/-Macrophage groups (Right) investigated (Lamin A+C: green and DAPI: blue). HA-0: PLGA/PLLA scaffold, HA-1: PLGA/PLLA scaffold with 1% w/v HA; HA-5: PLGA/PLLA scaffold with 5% w/v HA. Scale bars, 200 µm


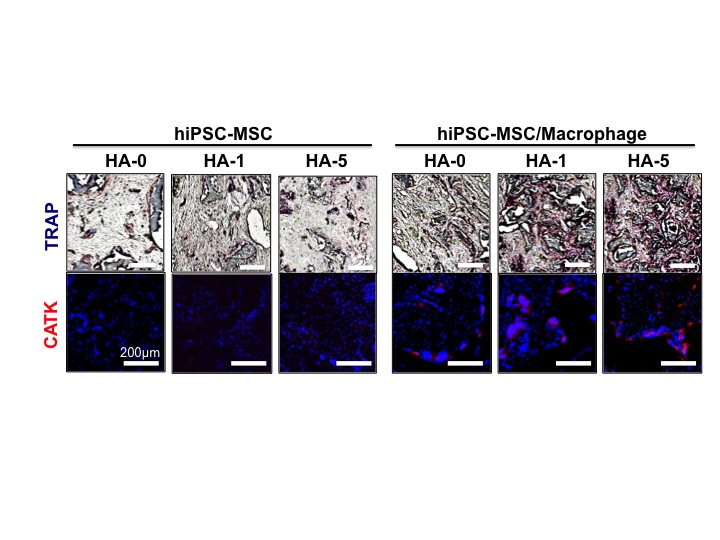


**Supplementary Fig. 4 Phenotypic and differentiation stability of hiPSC-macrophages to OCs in HA-based co-culture bone constructs *in vivo*.** Bone resorptive activity of OCs differentiated from hiPSC-macrophages was confirmed by TRAP and CATK staining of the implanted scaffolds after 8 weeks. HA-0: PLGA/PLLA scaffold, HA-1: PLGA/PLLA scaffold with 1% w/v HA; HA-5: PLGA/PLLA scaffold with 5% w/v HA. Scale bars, 200 µm

**Supplemental Tables**

**Table S1. Primers sequences used for RT-PCR**

| **Genes** | **Forward primers (5’-3’)** | **Reverse primers (3’-5’)** |
| --- | --- | --- |
| NFATC1 | TCTCAGGAGTGAAAGCATTGCACATA | AATGCTATGACCGAATGCAGCAGTTA |
| RANKL | CCAAGATCTCCAACATGACT | TAC ACCATTAGTTGAAGATACT |
| CATK | CAGTGAAGAGGTGGTTCAGA | AGAGTCTTGGGGCTCTACCTT |
| CTR | TCTCAGGAGTGAAAGCATTGCACATA | AATGCTATGACCGAATGCAGCAGTTA |
| TRAP 5b | TTCTACCGCCTGCACTCCAA | AGCTGATCTCCACATAGGCA |
| COL X | GGAATGCCTGTGTCTGCTTT | TGGGTCATAATGCTGTTGCC |
| COL I | GCTGGTCACCATGGTGATCAAGG | TACCAGGATGTCCAGTGCGAC |
| RUNX2 | CTTCACAAATCCTCCCAGTAGCTA | GGTTTAGAGTCATCAAGCTTCTGTCT |
| OCN | GTGACGAGTTGGCTGACC | TGGAGAGGAGCAGAACTGG |
| ALP | CACGGGCACCATGAAGGAAAA | ATTCTCTCGTTCACCGCCCAC |
| SOX-9 | GCATGAGCGAGGTGCACTC | TCTCGCTTCAGGTCAGCCTTG |
| Aggrecan | TGGGAACCAGCCTATACCCCAG | CAGTTGCAGAAGGGCCTTCTGTAC |
| C/EBPα | TCACCGCTCCAATGCCTA | CCTGCTCCCCTCCTTCTCTCA |
| FABPs | ACAGGAAAGTCAAGAGCACCATAA | TGACGCATTCCACCACCAGTT |
| LPL | GTCAGAGCCAAAAGAAGCAGC | GGGTTTCACTCTCAGTCCCAG |
| IL-6 | CCCCTGACCCAACCACAAAT | ATTTGCCGAAGAGCCCTCAG |
| IL-1β | GGACAAGCTGAGGAAGATGC | TCGTTATCCCATGTGTCGAA |
| TNFα | GGACAAGCTGAGGAAGATGC | TCGTTATCCCATGTGTCGAA |
| OPG | GCTAACCTCACCTTCGAG | TGATTGGACCTGGTTACC |
| OPN | GACACATATGATGGCCGAGGTGATAG | GGTGATGTCCTCGTCTGTAGCATC |
| β-actin | GCTCCTCCTGAGCGCAAGTAC | GGACTCGTCATACTCCTGCTTGC |
